# Investigating the impact of music therapy on two in-patient psychiatric wards for people living with dementia: retrospective observational study

**DOI:** 10.1192/bjo.2023.20

**Published:** 2023-02-23

**Authors:** Naomi Thompson, Kimberley Iyemere, Benjamin R. Underwood, Helen Odell-Miller

**Affiliations:** Cambridge Institute for Music Therapy Research, Anglia Ruskin University, UK; and Arts Therapies Service, Cambridgeshire and Peterborough NHS Foundation Trust, Fulbourn Hospital, Fulbourn, UK; Arts Therapies Service, Cambridgeshire and Peterborough NHS Foundation Trust, Fulbourn Hospital, Fulbourn, UK; Faculty of Science and Engineering, Anglia Ruskin University, UK; Older People and Adult Community Directorate, Cambridgeshire and Peterborough NHS Foundation Trust, Fulbourn Hospital, Fulbourn, UK; and Department of Psychiatry, University of Cambridge, UK

**Keywords:** Music therapy, agitation, distress behaviour, dementia, in-patient psychiatric wards

## Abstract

**Background:**

Music therapy can lift mood and reduce agitation for people living with dementia (PwD) in community and residential care settings, potentially reducing the prevalence of distress behaviours. However, less is known about the impact of music therapy on in-patient psychiatric wards for PwD.

**Aims:**

To investigate the impact of music therapy on two in-patient psychiatric wards for PwD.

**Method:**

A mixed-methods design was used. Statistical analysis was conducted on incidents involving behaviours reported as ‘disruptive and aggressive’ in 2020, when music therapy delivery varied because of the COVID-19 pandemic. Semi-structured interviews conducted online with three music therapists and eight ward-based staff were analysed using reflexive thematic analysis.

**Results:**

Quantitative findings showed a significant reduction in the frequency of behaviours reported as disruptive and aggressive on days with in-person music therapy (every 14 days) than on the same weekday with no or online music therapy (every 3.3 or 3.1 days, respectively). Qualitative findings support this, with music therapy reported by music therapists and staff members to be accessible and meaningful, lifting mood and reducing agitation, with benefits potentially lasting throughout the day and affecting the ward environment.

**Conclusions:**

We identified a significant reduction in the occurrence of distress behaviours on days with in-person music therapy when compared with no music therapy. Music therapy was reported to be a valuable intervention, supporting patient mood and reducing agitation. Interventional studies are needed to investigate the impact of music therapy and its optimum mode of delivery.

In 2019, it was estimated that 885 000 people in the UK were living with dementia, with 511 000 having advanced dementia, and these figures are expected to increase exponentially.^1^ The vast majority (90%) of people living with dementia (PwD) experience behavioural and psychological symptoms of dementia (BPSD), which can include agitation, depression, sleep disturbances, hallucinations, apathy and disinhibition.^2^ The authors acknowledge that the term BPSD does not accurately represent the experience of PwD, as distress behaviours can be caused by dementia and/or be expressions of unmet need. However, the term is used here to reflect the current literature and clinical language.^3^

In the UK, although there is an emphasis on supporting PwD in community or residential care settings, a minority, with the most advanced illness and BPSD, are cared for in National Health Service (NHS) in-patient psychiatric wards. Research evaluating care on these wards is extremely limited, as highlighted in a recent systematic review.^4^ However, the literature highlights the specialist nature of the work, caring for individuals who are unable to be safely cared for in other settings, with high rates of distress behaviours, termed ‘disruptive and aggressive behaviour’, on NHS wards.^5–8^ Use of sedative medication, predominantly antipsychotics, is common, but these drugs are associated with a number of significant adverse effects, including decreased cognition and increased risk of falls, stroke and death.^5,9^ This is accompanied by high rates of staff absence and use of agency staff.^5^ The impact on staff is important as research has long shown a close link between staff well-being, including job satisfaction and coping mechanisms, and patient well-being.^10,11^ However, very little research has investigated nonpharmacological interventions for this setting, despite calls for more research in this area.^12^

During the COVID-19 pandemic, service provision changed drastically. In-patient dementia wards in the UK experienced significant levels of mortality if the virus spread within them, and it was very difficult to contain an outbreak. As a result, efforts focussed on proactively minimising the number of people entering the wards and minimising leave. Thresholds for admission also increased during this time, as the risks from potential exposure to SARS-CoV-2 in an institutional setting had to be considered.

## Music therapy with PwD

Music therapy is a nonpharmacological, psychosocial intervention cited as a therapy that could support well-being in the National Institute for Health and Care Excellence guidelines for dementia.^13^ In the UK, music therapy is delivered by a Health and Care Professions Council (HCPC) registered music therapist on completion of a 2-year full-time, or 3-year part-time, Master's course. The therapist engages in predominantly live musical interactions with the client(s) to achieve established clinical goals. As outlined in previous research, music therapy may achieve benefit through multiple mechanisms, including arousal regulation, building interpersonal relationships, providing opportunity for nonverbal expression and reminiscence, empowerment and a form of person-centred activity.^14^ Research in community and residential care settings shows music therapy can reduce BPSD and support formal and informal caregivers.^15–19^ In addition, staff report that music therapy lifted mood for PwD and observing sessions enabled them to learn more about the PwD, thus enhancing their communication and relationship with individuals.^18^

In acute care settings for PwD around the world there is some evidence, including some clinical trials, to show that music therapy might reduce BPSD and levels of stress.^20–25^ Two studies have also shown short-term improvements in cognitive function following music therapy, with one study finding this to be most significant in the language subscale of the Mini-Mental State Examination.^21,26,27^ However, a small pilot, randomised controlled trial study found no significant difference between individual music therapy and an active engagement intervention, involving simple occupational activities delivered by a social worker, when measuring reduction in BPSD.^28^ None of these studies investigated staff perception of the interventions delivered. Although research into music therapy on NHS in-patient psychiatric wards for PwD is limited, implementation of the interventions appears to be feasible and an increase in positive, and reduction in negative, behaviours has been described on days when it was delivered, as defined by the authors.^29–31^ One study reported staff feedback, collected through questionnaires. The feedback highlighted the impact of group music therapy on patient well-being and engagement, and the enhancement of staff–patient and staff–staff relationships.^30^ A recent audit of music therapists working on NHS in-patient wards for PwD showed few music therapists work in this setting, although there were similarities in practice and respondents felt valued by the multidisciplinary teams they worked with.^32^

During the COVID-19 pandemic in the UK, many in-person music therapy interventions moved online. Research on music therapy groups with PwD living at home in the community suggest that these groups provided an important space for social interaction and emotional connection, although some participants had difficulties accessing the online space.^15,33^ To date, no literature has explored the impact of music therapy interventions delivered online with PwD within a residential or in-patient setting.

This study was planned and conducted during the COVID-19 pandemic, enabling us to draw upon the unique changes in intervention delivery that became necessary in the UK at the time, as outlined above. Through this opportunistic research, we seek to advance understanding of whether music therapy may be a helpful intervention on in-patient psychiatric wards for PwD, with a focus on early indications of any impact on incidents of distress behaviours and staff perceptions of the intervention. The following research question was established: What are the likely benefits of weekly group music therapy on NHS in-patient psychiatric wards for PwD?

To answer this, the following research objectives were outlined: (a) to evaluate the difference in the number of incidents involving distress behaviours, reported on wards as disruptive and aggressive behaviour, on days with in-person, online or no music therapy; and (b) to investigate staff perceptions of the impact of music therapy for PwD on in-patient psychiatric wards.

## Method

A mixed-methods study was designed to investigate whether music therapy, delivered as part of standard care, could be a helpful intervention on in-patient psychiatric wards for PwD. Changes in incident rates of distress behaviours, recorded on wards as disruptive and aggressive behaviour, were observed retrospectively on days with and without music therapy. The perceived impact of music therapy was investigated through semi-structured interviews with members of the multidisciplinary team. The team presented and discussed the project with experts-by-experience engaged in a dementia patient and public involvement group at Anglia Ruskin University. The views of patients were not included in this study as the quantitative element was an opportunistic data-set arising from the unique circumstances of a viral pandemic, rather than a piece of work planned in advance. This group of patients are a specific and small subset of patients with dementia, and it is important that patient views are relevant to in-patient care. As a result, the authors have worked with colleagues and Dementia UK to create a bespoke patient and public involvement and engagement group for in-patient dementia care, who are working closely with the authors to develop future research, although this was not available when the project was conceived.

Data were gathered from two in-patient psychiatric wards for PwD (referred to as ward 1 and ward 2), situated on different sites in one NHS Trust. These wards care for PwD in the region experiencing the most significant distress, with patients admitted if they cannot safely be cared for in community or residential care settings, often using the legal framework provided by the Mental Health Act.^34^ Ward 1 has 14 beds. Ward 2 has eight beds for PwD and 14 beds for older people with functional illness. During COVID-19 lockdowns these sections were cared for together, so quantitative data relates to all patients on ward 2. Usual care on both wards includes a range of group and individual activities organised by the occupational therapist and occupational therapy assistant. Activities include music, cooking, art activities and walking, depending on the interests of the patients. Family members and loved ones can visit regularly; however, all visits were suspended during lockdowns in the UK, with exceptions for end-of-life care. Both wards have a part-time medical consultant, nurses, healthcare assistants, occupational therapists and therapy assistants. They also receive a weekly open music therapy group, delivered by an HCPC-registered music therapist. Other professionals, such as physiotherapists, speech and language therapists, arts therapists and psychologists, are available to support individuals on referral.

The authors assert that all procedures contributing to this work comply with the ethical standards of the relevant national and institutional committees on human experimentation and with the Helsinki Declaration of 1975, as revised in 2008. Ethical approval was provided by the School Research Ethics Panel at Anglia Ruskin University. Analysis of retrospectively collected data from two in-patient NHS wards was conducted as a service evaluation, with approval provided by the quality assurance and clinical effectiveness department at the Cambridgeshire and Peterborough NHS Foundation Trust. Additional ethical approval to conduct interviews with staff was obtained through the Integrated Research Application System (IRAS) (project identifier 301062). The study was registered with the Cambridgeshire and Peterborough NHS Foundation Trust Quality Improvement Office.

### Observational data of rates of incidents on the ward and their relationship to music therapy provision

Incidents reported on the NHS Trust's secure, internal reporting system, Datix (RLDatix, London), in the calendar year 2020 were gathered retrospectively. Datix is the electronic risk reporting and management system in use in the NHS at the time of writing. Staff complete a Datix incident form in the NHS Trust in question if they are aware of any incident that results in harm to patients or staff, or could cause harm in the future. At the time of reporting, staff assign the incident to a category, e.g. ‘accident’, ‘clinical assessment and treatment’ or ‘disruptive and aggressive behaviour’. All incidents have a primary manager, but are also tracked and scrutinised by a Trust-wide patient safety team, as well as being centrally reported. All data were anonymous at the point of collection. A list of dates that music therapy was delivered on the ward and the method of delivery, in-person or online, was provided by the music therapist. Data were separated to two time periods – before lockdown was initiated and after the ward went into lockdown – to reflect the difference in service provision during these times. Because of the opportunistic and retrospective nature of the data collection, it was not possible to gather data about the individual patients on the wards during the data collection period.

### Statistical analysis

Statistical analysis was conducted by the lead author with IBM SPSS Statistics for Windows version 26, and reviewed by a statistician external to the research team.^35^ Descriptive statistics were conducted on the whole data-set to establish the frequency of all reported incidents. Additional analysis was conducted by looking at the reporting rate of incidents recorded as disruptive and aggressive behaviour on days with in-person, online or no music therapy. Disruptive and aggressive behaviour, also known as distress behaviour, which occurs in more than 40% of cases, can include shouting, grabbing, pushing, hitting, kicking and spitting. Only days where music therapy would usually have been delivered (had COVID-19 restrictions not been in place) were included in this analysis. A one-sided *t*-test with equal variance was conducted to test the statistical significance of the difference in rates of incidents between the conditions, with a significance level of *P* < 0.05.

### Semi-structured interviews

All members of the multidisciplinary team working on two wards for PwD were invited for interview by email, with agreement and support from the ward managers. This included allied health professionals (such as other arts therapists, occupational therapists, physiotherapists, and speech and language therapists), healthcare assistants, nurses and doctors. Staff were invited regardless of the length of time they had been in employment on the ward or their involvement in the music therapy group. As only a small number of individuals were eligible to participate (staff members working on two in-patient wards), no additional sampling method was used. In addition, three music therapists who had delivered interventions on the wards were invited to participate in the interviews. All participants provided written consent.

The interviews were semi-structured, following a prepared topic guide including a list of topics with accompanying prompts, allowing flexibility to explore additional content that arose. The topics were designed from the literature review and outcomes of a survey of music therapists working in NHS in-patient psychiatric wards for PwD.^32^ Although the structure of the interviews was the same, topic guides for music therapists and other members of the multidisciplinary team were adjusted to reflect their differing roles (Table 1).

**Table 1:** Interview topics provided to music therapists and other members of the multidisciplinary team before interview

Topics for music therapists	Topics for other staff members
Structure of the music therapy provision and theoretical approaches Observed benefits and value of music therapy Role of staff and family members Challenges to delivering music therapy in this setting	Knowledge and understanding of music therapy Observed benefits and value of music therapy Role of other staff in the music therapy service Challenges to working in this setting

Interviews were conducted online by the first author. Volunteers who came forward within the agreed 4-month collection period were invited to participate, after which point data collection stopped. Because of the small number of staff members eligible, no limit was placed in advance on the number of participants. Interviews lasted between 30 and 60 min, according to how much material participants discussed. Interviews were recorded and transcribed using the programmes’ internal software, with a backup recording on a separate secure device. Transcriptions were edited by the interviewer to check for accuracy. Verbatim transcription was used, recording ‘uhms’ and repeated words, although laughter and background noise was not noted. Where quotes are included in the results section, ‘uhms’ and repeated words have been removed for ease of reading.

Reflexive thematic analysis was conducted by the first author, who was external to the NHS employed staff team, and reviewed by the last author, who is very experienced in qualitative analysis.^36,37^ Both authors are researchers and music therapists with experience working with people with dementia. Six stages of analysis were conducted: (a) familiarisation with the transcript, (b) inductive coding of each transcript, (c) codes were grouped under subthemes within each transcript, (d) subthemes within transcripts were grouped and combined to create subthemes across all transcripts, (e) subthemes across responses were grouped into overarching themes and (f) the relationship between the overarching themes was explored and supporting quotes were identified.^37^ Analysis was conducted separately for responses from music therapists, and those from other staff members. When all stages were complete, relationships between data in these separate groups were explored, with similarities and differences identified.

### Music therapy intervention

As this project gathered data retrospectively, the music therapy service offered to the wards during the collection period reflected usual practice. This involved a weekly open music therapy group delivered by the same therapist on both wards between 13.00 h and 14.00 h on the same weekday each week (i.e. Tuesday on ward 1 and Friday on ward 2). The timing was chosen in collaboration with the multidisciplinary team to encourage group interaction after lunch. The groups took place in communal areas and all patients were welcome and encouraged to take part, along with any family members and staff present. Activities included singing preferred familiar songs supported by the music therapist on piano or guitar; playing tuned and untuned percussion instruments; activities to support cognition, such as turn-taking and encouraging focused attention; and light physical exercise.

Because of the COVID-19 pandemic, all in-person therapy interventions were stopped on 13 March 2020. From 28 April 2020 an online music therapy open group was established on ward 1. The music therapist used similar techniques to in-person sessions, with a focus on familiar songs to initiate engagement before introducing interactive musical activities if appropriate. Discussion about the songs and the memories these provoked was encouraged. It was not possible to arrange online music therapy on ward 2. The authors were mindful that staff were providing care to a very vulnerable patient group in the context of a global pandemic, where there were limited time and resources for engaging in new modes of service delivery. In-person music therapy had resumed on both wards at the time of the staff interviews (August to October 2021).

## Results

### Impact of music therapy on rates of incidents of distress behaviours

The average length of stay for both wards during 2020 was 85.81 days. A total of 413 incidents were reported between 1 January and 31 December 2020 across the two wards. Distress behaviours, reported on site as disruptive and aggressive behaviour, were the most reported incidents on both wards, accounting for 46.5% (131) of incidents on ward 1 and 53.4% (70) of incidents on ward 2.

For days when music therapy was scheduled to take place, an in-person session was delivered on 14 (10%) days, an online session on 28 (20%) days and the group was cancelled on 98 (70%) days. Cancellations were predominantly attributable to the COVID-19 pandemic, with occasional breaks when the music therapist was on leave. A total of 86 incidents were recorded on these days, 44 (51%) of which were reported as disruptive and aggressive behaviour.

**Fig. 1:**
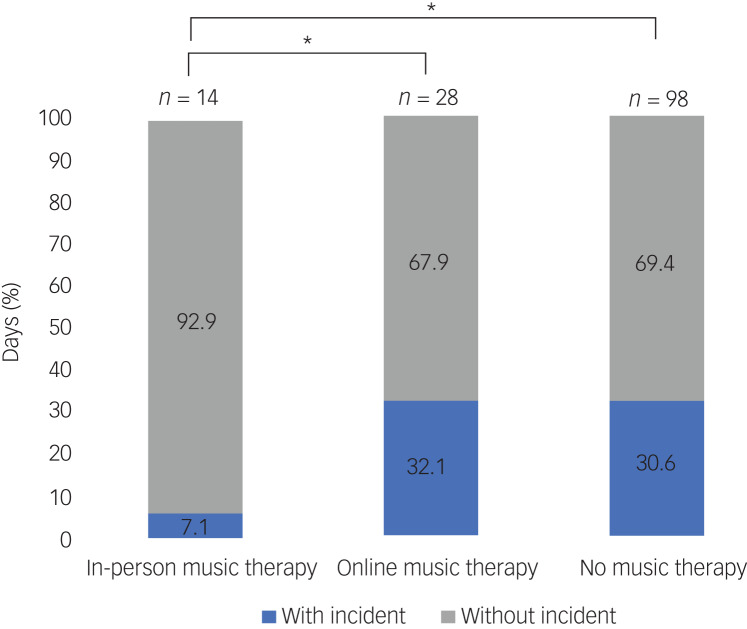
The percentage of days on which an incident of disruptive and aggressive behaviour was reported when in-person, online or no music therapy was delivered. Statistically significant relationships (*P* < 0.05) between conditions are indicated with an *, calculated using a one-sided *t*-test with equal variance.

On average there was an incident of reported disruptive and aggressive behaviour every 14 days when music therapy was delivered in-person, every 3.1 days when music therapy was delivered online and every 3.3 days when no music therapy was delivered. This was equivalent to an incident on 7.1% of days with in-person music therapy, 32.1% of days with online music therapy and 30.6% of days with no music therapy (Fig. 1). The number of incidents on days with in-person music therapy was significantly lower than the number of incidents on days with online music therapy (*P* = 0.038) and no music therapy (*P* = 0.034) (Fig. 1). The difference in the number of incidents on days with online and no music therapy did not reach significance (*P* = 0.439).

### Staff perceptions of the impact of music therapy on psychiatric in-patient wards for PwD

A total of 11 interviews were conducted, with three music therapists, six staff from ward 1 and two staff from ward 2. Staff roles included occupational therapists (two), occupational therapy assistants (two), healthcare assistants (two), a junior doctor (one) and a consultant doctor (one). No nursing staff volunteered to interview. Length of staff employment on the ward ranged from 2 months to 30 years. Staff were involved in the groups in a variety of ways, from supporting the running of the group to observing the sessions with the option to join if available. Staff responses came under three themes, with seven subthemes (Table 2).

**Table 2:** Themes, subthemes and codes from semi-structured interviews with music therapists and staff members on two in-patient psychiatric wards for people living with dementia

Themes	Subthemes	Codes
Music therapists and staff perceived music to be accessible for PwD	Music reported to enable PwD to access the well-self	Innate, neurological basis to music that was thought to transcend language barriers, enabling all to engage in interactions regardless of impairment
		Individualised music, often from childhood, perceived to help PwD access memories and express emotions, often bringing joy
Music is used by music therapists and staff to interact with patients	Music therapists respond flexibly to the environment, which supports engagement	The music therapist uses live familiar songs, improvisation, instrumental playing and music-listening flexibly, following the patient's lead
		Music therapy reported to meet a need for meaningful, accessible therapeutic activity where patients cannot engage in talking therapies^a^
		Individual, open and closed groups can be delivered to meet different aims^b^
		Music therapists observation skills help them regulate arousal and maintain safety, which might be different to other musicians^b^
	Collaboration with staff and family members supports delivery of music therapy	Staff support with the practical running of sessions and provide one-to-one support within the group
		Music therapists learn about the patient through discussions with family members and staff^b^
		Collaboration is possible as staff see the benefits of music therapy^b^
	Staff use predominantly recorded music intentionally and unintentionally	Staff use individualised recorded music and singing, with family member's support, to facilitate planned and spontaneous group and individual interactions^a^
		Recorded music played in the background without intent to effect change^a^
Music therapy reported to have a positive impact for patients and the wider ward environment	Music therapy is perceived as having a positive impact for patients before, during and after sessions	Music therapy brings most patients together as they are able to engage actively and/or listen, helping patients feel connected
		Music therapy is a highlight of the week: lifts mood and calms agitation, with effects observed before group and often lasting for the rest of the day
		Music therapy is seen to create equality, with those who are withdrawn and those who are agitated being empowered to participate and lead
		Online sessions perceived as less engaging than in-person sessions, but provided continued support
		In music therapy, staff see a side of patients that is not seen anywhere else^a^
		No known negative effect of music therapy, with patients able to leave if they choose to^a^
		Sharing memories can help patients feel valued and heard^b^
	Music therapy is part of creating an enriching environment in a place that can be challenging and aggressive	Indirect effect for staff as their job is easier if patients are happy
		Staff are often dealing with instrumental or challenging tasks Levelling and combining for all to engage in positive activity together
		Joy for staff to see patients engaged and calm in music therapy, which affects their relationship and understanding of how to support patients^a^
		Music therapy can support staff to use music outside of sessions^b^
	More music therapy would allow for more targeted interventions, but greater knowledge of music therapy is needed	More music therapy would allow for individually tailored interventions and contribution to care plans and multidisciplinary team meetings
		All staff are keen for more groups using familiar songs and open to family members^a^
		Some uncertainty about the ways in which music therapy could be delivered, as staff have only experienced groups and do not know the evidence base^a^

Responses were common across both participant groups, unless otherwise indicated. PwD, people living with dementia.

a. Staff member responses only.

b. Music therapist responses only.

#### Music therapists and staff members perceived music to be accessible for PwD

Both music therapists and staff members reported that music is an important medium for PwD. The way music interacts with the brain was thought to transcend verbal language, enabling people to engage and communicate regardless of their cognitive impairment. Music was also perceived to help patients access memories, especially from their childhood and young adult years, which brought joy and an opportunity for emotional expression. Respondents felt that these elements of music enabled PwD to access their preserved abilities, referred to by some participants as the ‘well-self’, when engaging in musical activities.

Examples of participant responses include:

‘Across a huge range of cognitive impairments, people can relate to music. I think there's something deep within everyone's minds that can relate to music’ (P7, staff member).

‘I just think everybody likes music. It makes you feel good, doesn't it. I think like I said before, often with older people, it evokes memories’ (P10, staff member).

#### Music is used by music therapists and staff to interact with patients

Music was seen as an important part of ward life, with live music being used by music therapists in clinical sessions and planned and spontaneous musical interactions between staff members and patients occurring daily. Live musical interactions were reported by both music therapists and staff to enable the music therapy group to flexibly follow the patients’ lead. They also stated the importance of close collaboration between the music therapist and staff members for the successful running of the group. Staff members commented that music therapy met a need for meaningful therapeutic activity in a setting where few patients could engage in talking therapies. Music therapists discussed how they use their observation skills in sessions to maintain safety, particularly monitoring levels of arousal, which was thought to be a difference between sessions delivered by trained music therapists and by other musicians.

Examples of participant responses include:

‘I mean that's the lovely thing about music, that you can flow from one kind of place to another and quite easily steer things around a bit’ (P2, music therapist).

‘[the music therapist] comes onto the ward, sort of runs the session herself, but we will sort of facilitate that in getting instruments and things that we have on the ward and then actually being in that session and sort of sitting next to some of the patients and helping them to engage with it’ (P11, staff member).

Staff members also talked about the intentional and unintentional use of music outside of the music therapy sessions. Staff ran planned music sessions using familiar recorded music, with handheld percussion available, as well as having more spontaneous musical interactions with patients by using both recorded music and singing. Staff mentioned that recorded music, such as the radio, could be played in the ward without intention to impact the ward environment.

Examples of participant responses include:

‘Yeah, they're more like sing-a-longs, yes, and we got musical instruments so sometimes patients will choose a musical instrument, or sometimes they'll say “Oh no”’ (P9, staff member).

‘It's always better if a person is doing it, than the actual radio. They interact more and it's more a personal thing’ (P6, staff member).

#### Music therapy was reported to have a positive impact for patients and the wider ward environment

Staff members and music therapists reported that the music therapy lifted mood and calmed agitation, beginning during the set-up for the session and often lasting for the rest of the day. It was felt that the ability for patients to engage in the group actively, through singing, playing instruments, and/or dancing, or to sit and listen to the music, enabled more patients to engage and created a feeling of connection. There was also equality between patients who were more often withdrawn and those who presented as distressed, as all were empowered to interact and lead. Staff members reported that they saw a side of patients in music therapy that they did not see anywhere else, and that they had never seen a negative effect of the group. However, music therapists and staff agreed that online sessions, while providing some continued support during the pandemic, were not as engaging or effective as in-person sessions.

Examples of participant responses include:

‘I think that would be true to say for a lot of people, that having had that positive experience, that carries on for them for the rest of the day’ (P4, staff member).

‘For that moment there's a real kind of positive interaction, or you're making a difference there. Whether that's reducing anxiety. Whether that's just someone feels connected with another person for that moment’ (P2, music therapist).

The music therapy group was seen as an integral part of creating an enriching environment within a setting where it can be hard to find appropriate nonpharmacological interventions to support patients. There was an indirect positive impact reported for all staff, as many patients were engaged in a meaningful activity in the moment and therefore were less likely to be displaying distress behaviours. This calmer atmosphere potentially lasted for the rest of the day. For staff members who interacted in the group, either actively or through observing, it was seen as helpful and levelling to engage positively with patients in a way that was different to their usual instrumental tasks. Staff reported that it was a joy to see patients happy and calm in the session, and that this helped them learn about the patient and how they could support them outside of the group.

Examples of participant responses include:

‘If the patients are happy it makes our job easier’ (P5, staff member).

‘[Be]cause I think music can be great at sort of binding people together’ (P11, staff member).

Both music therapists and staff members felt that more music therapy would be helpful, allowing for individually tailored interventions which could be incorporated into the individual care plan and shared in multidisciplinary team meetings. However, some staff members, although keen for more groups, were unsure about the other ways music therapy could be used on the ward as they had not experienced these.

Examples of participant responses include:

‘I would like to see both [group and individual music therapy] because sometimes it worked with a group, but when you need it for one person and they get so much out of it and nothing else works, music usually works, so that's lovely’ (P6, staff member).

‘I really do believe that it makes such a massive impact that if we could have more, the more the better, definitely, without a doubt’ (P8, staff member).

## Discussion

The findings of this mixed-methods observation study suggest that music therapy could be a helpful intervention on in-patient psychiatric wards for PwD, with the potential to lift mood and calm agitation and, possibly, reduce associated distress behaviours. Quantitative analysis of reported incidents showed a significant reduction in reporting rates of disruptive and aggressive behaviour on days with in-person music therapy than on days with no music therapy. The qualitative data support this, with music therapy identified by music therapists and ward staff as an accessible and meaningful intervention for PwD, lifting mood and calming agitation, which appears to have a positive impact on the wider ward environment.

To our knowledge, no previous studies have looked at the impact of music therapy on NHS in-patient psychiatric wards for PwD through collecting both qualitative and quantitative data and exploring staff perception of the intervention. Our study fills this gap in the literature, presenting novel findings that further understanding of the impact of music therapy practice in this setting, which can inform future research. In particular, we were able to use the changes in therapy provision in response to the COVID-19 pandemic, to investigate the impact of music therapy on rates of distress behaviours.

Incidents involving behaviours reported to be disruptive and aggressive were the most reported incidents on both wards during 2020, a problem that has been identified in studies looking at multiple NHS in-patient psychiatric wards for PwD.^5^ The reduction in rates of distress behaviours on days with in-person music therapy shown here support other studies looking at the impact of group music therapy in in-patient psychiatric settings for PwD, which identify a positive impact on BPSD.^20–22,29,31^ However, previous studies of music therapy in this setting have not examined the perception of staff and the impact on the ward environment. We found that staff experienced music therapy as having a direct and indirect positive impact on staff, and expressed a need for greater access to the interventions and understanding of additional ways music therapy could be used to meet patient need. An inherent understanding of the role of music to enable positive interactions with PwD was also shown, as staff played recorded music in planned and unplanned ways on the ward. Despite these positive findings, a recent audit showed that few wards in the UK have access to music therapy, suggesting that there may be barriers to accessing this intervention in this setting.^32^

Antipsychotic medication used to treat distress behaviours have significant adverse effects, including increased mortality.^9,10^ Calls for increased implementation of nonpharmacological interventions have been made. Our findings suggest that music therapy may be an effective nonpharmacological treatment for BPSD, with a significant reduction of distress behaviours on days of in-person intervention delivery shown, supported in the qualitative data, with lasting changes to patient mood and levels of agitation reported by music therapists and staff members. This concurs with similar findings suggesting that music therapy reduces levels of BPSD in residential care settings.^18,19^ There are a number of factors that could contribute to this. First, studies measuring biomarkers during music therapy groups on in-patient wards for PwD suggest that patients were less stressed during groups, although changes were not found to be long lasting.^21,23–25^ It may be that engaging in nonverbal, person-centred and accessible therapeutic activities lowers stress and improves mood, thus supporting patients to feel more calm and be less likely to display distress behaviours. In addition, the impact of working alongside staff to identify and address unmet needs on reducing violent incidents on older people's mental health wards has been reported, with a 20% decrease in incidents reported as violent.^6^ As the music therapy group was reported to provide staff with an opportunity to engage in a meaningful, positive interaction with patients, this may have affected care delivery outside of the group.

The impact of music therapy warrants further investigation into how the intervention could be optimised to meet patient need in these settings, and support staff and family members. Music therapy interventions and services for in-patient psychiatric wards for PwD should draw on developments in the community and residential care settings for PwD, with a focus on providing healthcare staff and family members with the skills to embed musical interactions in everyday care to intentionally reduce BPSD and isolation.^14,15,18^ The clinical and cost effectiveness of a music therapy provision with additional time on a ward to deliver training alongside clinical sessions has similarly not been investigated. Future research should measure the impact of an embedded music therapy programme on use of antipsychotic medication, rates of distress behaviours, BPSD, patient length of stay, discharge destination, staff job satisfaction, sick leave and well-being. This would need to include the measurement of positive outcomes, such as patient, staff and relative quality of life, through standardised questionnaires and qualitative methodologies. To this end, the research team are developing a co-design project to co-develop an embedded and costed music therapy intervention, together with experts-by-experience and stakeholders, and pilot this on in-patient psychiatric wards for PwD to measure feasibility, acceptability and helpfulness. The impact of the intervention on patient, staff and relative well-being and ward outcomes could then be measured in controlled trials.

### Limitations

This was a retrospective observational study, with a small sample, with data gathered from two wards within one NHS Trust, so results are not necessarily generalisable to other in-patient psychiatric wards for PwD. There could be bias as the analysis was conducted by a researcher working within Anglia Ruskin University who is also a practicing music therapist, although not within the NHS Trust in question. Attempts to mitigate this through checking analysis with a statistician external to the research team were made. Because of the naturalistic design of the study, quantitative data were gathered retrospectively from the NHS Trust's internal reporting system. Therefore, other factors that could have influenced the prevalence of distress behaviours, such as staffing levels and the delivery of other interventions, were not controlled for. In addition, although Datix captures a range of incidents, it will not have captured all distress behaviours. Further research involving detailed observational methodology and increasing staff reporting are required to accurately capture relevant incidents. Changes to practice owing to the COVID-19 pandemic, including combining the care for PwD and those with functional illness on one of the wards, could have significantly affected the data. Also, the frequency of behaviour reported as disruptive and aggressive could have been higher during periods of lockdown because of visitation restrictions and potential effects on staffing levels and morale. To mitigate this, data were collected from across a calendar year and were separated according to the type of restrictions the ward was under at different times in the year. Also, when analysing rates of incidents on days when music therapy was or was not delivered, weekdays included in the analysis were kept consistent and Saturdays and Sundays were not included because fewer activities take place on weekends, which could have skewed the data. A much larger data-set, including multiple wards, is needed to provide robust findings. However, this is challenging as music therapy is not available on all dementia in-patient wards in the NHS, and the delivery of the intervention is not standardised.

There are also limitations to the qualitative data collection and analysis. The sample is small and there is a potential for interviewer bias and volunteer bias. As the interviewer is a music therapist (see above), they may be biased toward the positive impact of music therapy. Staff members who volunteered may have been those with more interest or support for the music therapy interventions. Attempts to mitigate this were made through designing a topic guide and a clear protocol for the use of reflexive thematic analysis, in collaboration with the research team. Also, the team strove to interview staff from a range of positions in the multidisciplinary team across both wards, although unfortunately no nurses volunteered to participate despite nursing ward managers being supportive of the research. Further research, including additional time for data collection, reimbursement of staff time and data-checking with staff, is needed to validate these findings.

In conclusion, findings from this observational mixed-methods study suggest that music therapy is associated with decreased rates of distress behaviours, and is viewed favourably by staff working on in-patient dementia wards in psychiatric hospitals. Music was seen as important for PwD, and staff showed a commitment to using preferred music to initiate positive interactions with patients. Future research should identify barriers to implementing music therapy in this setting, the impact of different modalities and delivery, and the impact of training for staff and family members to deliver music-based interventions. Data from observational studies such as this one are encouraging, but a move to rigorously evaluated interventional studies is now required to understand the nature and extent of any benefit from music therapy, a treatment that has the promise to bring significant benefit to a patient group experiencing significant distress and where current treatment is limited.

## Data Availability

No study data are available so as to protect the identity of patients and participants in the study.
